# INTERESFINCTERIAL LIGATION OF FISTULA TRACT (LIFT) FOR PATIENTS WITH ANAL FISTULAS: A BRAZILIAN BI-INSTITUTIONAL EXPERIENCE

**DOI:** 10.1590/0102-6720201700040002

**Published:** 2017

**Authors:** Sérgio Eduardo Alonso ARAÚJO, Marcelli Tainah MARCANTE, Carlos Ramon Siveira MENDES, Alexandre Bruno BERTONCINI, Victor Edmond SEID, Lucas Araujo HORCEL, Rodrigo Oliva PEREZ, Sidney KLAJNER

**Affiliations:** 1 Coloproctology, Hospital Israelita Albert Einstein, São Paulo, SP;; 2 Coloproctology, Santa Izabel Hospital, Salvador, BA;; 3 Instituto Angelita & Gama, Coloproctologia, São Paulo, SP, Brazil

**Keywords:** Rectal fistula, Abscess, Anus., Fístula retal, Abscesso, Ânus

## Abstract

*****Background*** ****:**

The best treatment for anal fistula should extirpate infection and promote healing of the tract, whilst preserving the anal sphincter complex and full continence.

***Aim*:**

To analyze the success rate after a modified technique for ligation of the intersphincteric fistula tract (LIFT) for patients with anal fistulas.

***Methods*:**

A prospective (observational cohort study) Brazilian bi-institutional experience with a modified (ligation of the intersphincteric fistula tract without excision) LIFT technique was undertaken. A clinical database was settled for the following variables: age, gender, BMI, comorbidities, distance between external orifice and the anus, previous fistula surgery, type of fistula, operative time, intra- and postoperative complications, duration of follow-up, and success rate.

***Results*:**

Between November 2015 and January 2017, 38 patients with transsphincteric fistulas were operated on using the modified LIFT procedure. Seventeen (44.7%) were men. Median age was 41 (18-67) years. Median BMI was 26.4 (22-38) kg/m^2^. Five (13.2%) had undergone previous surgery. The fistula was transsphincteric in all cases. Median follow-up was 32 (range, 14-56) weeks. Success was observed in 30 (79%) patients.

***Conclusions*:**

The LIFT technique without excision of the fistula tract proved to be safe and effective for transsphincteric anal fistulas.

## RESUMO

*****Racional*** ****:**

O melhor tratamento para a fístula anal deve eliminar a infecção e promover a cicatrização do trato, preservando o esfíncter anal e a continência completa.

***Objetivo*:**

Determinar a taxa de sucesso após o uso da técnica modificada de ligadura interesfincteriana do trato fistuloso (LIFT) para pacientes com fístulas anais.

***Métodos*:**

Estudo de coorte observacional brasileiro bi-institucional com o procedimento LIFT modificado (ligadura interesfincteriana do trato fistuloso sem excisão). Foi estabelecida base de dados clínica para as seguintes variáveis: idade, gênero, IMC, comorbidades, distância entre o orifício externo e o ânus, operação anterior para fístula, tipo de fístula, tempo cirúrgico, complicações intra e pós-operatórias, duração do seguimento e taxa de sucesso.

***Resultados*:**

Entre novembro de 2015 e janeiro de 2017, 38 pacientes com fístulas transesfincterianas foram operados com o procedimento LIFT modificado. Dezessete (44,7%) eram homens. A idade média foi de 41 (18-67) anos. O IMC médio foi de 26,4 (22-38) kg/m^2^. Cinco (13,2%) tinham sido submetidos à operação anterior. A fístula era transesfincteriana em todos os casos. O acompanhamento médio foi de 32 (14-56) semanas. Sucesso foi observado em 30 (79%) pacientes.

***Conclusões*:**

A técnica LIFT sem excisão do trato da fístula provou ser segura e eficaz para fístulas anais transesfincterianas.

## INTRODUCTION

An anal fistula is a persistent infectious tract developing between the anal canal and the perianal skin. Most commonly due to a cryptoglandular infection, it appears after drainage of a perianal abscess, although other causes include Crohn’s disease, trauma, radiation, or malignancy^1^. Complex fistulas include high transsphincteric (>30% external sphincter involvement), suprasphincteric, or extrasphincteric fistulas. They may also be defined as horseshoe, recurrent, and all anterior fistulas in women and may also present with multiple tracts, or associated to Crohn’s disease, radiation therapy, or malignancy^2^. 

The best treatment for anal fistula should extirpate infection and promote healing of the tract, whilst preserving the anal sphincter complex and full continence. For non-complex more distal cases, surgical options, such as lay-open of the fistula tract, are very effective with a success rate of up to 100%^3^ and with incontinence rates under 10%^4^ ^,^ ^5^. 

Regarding complex fistulas, a clinical consensus about the best operation is still lacking. No single technique has been shown to be suitable for all cases. Furthermore, recurrence rates after surgery seem to be high although studies with proper follow-up are scarce. Therefore, complex anal fistulas management remains a challenge for surgeons worldwide^6^. The ligation of the intersphincteric fistula tract (LIFT) procedure has been described as the most recent innovation to sphincter-preserving surgery for the management of complex anal fistulas. It was first reported by Rojanasakul et al.^7^ in 2007 as a sphincter-preserving procedure, primarily indicated for transsphincteric fistulas. These authors hypothesized that the ligation and excision of the intersphincteric portion of the fistula tract would close the internal opening, as well as eliminate the septic focus. Since the sphincter muscle is not divided, the impact on continence is presumably negligible. In their initial report of 18 patients, a 94% success rate with no changes in continence was observed. Given these promising initial outcomes, the LIFT procedure has gained tremendous popularity among surgeons. 

Following the description of the technique by Rojanasakul et al.^7^, several studies have been published on the LIFT procedure, with success rates varying from 47-95%^8^ ^-^ ^10^. Interestingly, current experiences have reported success rates under 50%^11^ ^,^ ^12^. Factors that can adversely affect LIFT results have been reported in an inceptive manner. It remains controversial if the number of previous fistula surgeries may^13^ or may not^11^ affect success rates. Moreover, Liu et al.^14^ have suggested that a fistula tract of less than 3 cm was associated with a higher healing rate. In addition, other authors have advocated deferring the LIFT procedure until local sepsis is solved through the insertion of a drainage seton^11^ ^,^ ^15^. Different results may be due to the inclusion of technical variations and different presentations of anal fistula disease may play a role. Ultimately, the systematic reviews^2^ ^,^ ^16^ ^-^ ^18^ evaluating cumulative evidence about LIFT have reported solely on the pooled success rates, including all the technical variations. Therefore, when we look at published LIFT procedure results for the cure of anal fistula, it remains challenging to define which patients will benefit most. 

In the present study, it was aimed at evaluating the success rate after a modified LIFT procedure for the surgical treatment of complex anal fistulas without previous seton drainage. 

## METHODS

The present paper reports a prospective observational bi-institutional cohort study. The primary endpoint was success after surgery for anal fistula using the LIFT procedure. Success after the LIFT procedure was defined as complete healing of the surgical intersphincteric wound and the external opening without any sign of recurrence. Failure was defined as a clinical diagnosis of fistula recurrence at any time in the postoperative follow-up defined by clinical interview, physical examination, and MRI (magnetic resonance imaging). 

The present study underwent Institutional Review Board approval at the two institutions. A prospective analysis of consecutive patients with anal fistulas undergoing surgical treatment using the LIFT procedure between November 2014 and November 2015 was conducted. No randomization was performed. Written informed consent for the LIFT procedure and agreement to participate in regular follow-up assessments were obtained for all included patients.

Inclusion criteria were all complex cryptoglandular anal fistulas (transsphincteric or suprasphincteric) in patients with newly diagnosed anal fistula as well as in those who had undergone previous attempts at fistula repair. Patients were referred for MRI scans at the discretion of the attending surgeon. Exclusion criteria were superficial fistulas that could be treated by a simple fistulotomy (lay open) procedure, patients with anal fistulas associated with suspected or confirmed Crohn’s disease, previous radiation therapy and colorectal malignancy. 

A clinical database was created to collect information regarding all patients included in the present series. Collected variables were: age, gender, BMI, comorbidities, distance between external orifice and the anus, previous fistula surgery, type of fistula, operative time, intra- and postoperative complications, duration of follow-up, and success rate.

### Surgical technique

In the present study, all patients were operated on using the LIFT technique without excision of the remnant of the fistula tract between the ligation and the internal orifice as described by Rojanasakul et al.^7^.Patients received no bowel preparation or enemas. Antibiotic therapy started 1 h before surgery and lasted 24 h. Patients were operated on in the lithotomy position under spinal anesthesia combined with intravenous sedation.

Previous seton drainage was not used in any case. The fistula tract was clearly defined and the internal opening was identified by using a probe or through hydrogen peroxide injection into the external fistula opening. A small (2-3 cm) curvilinear incision was then created over the intersphincteric groove at the level of the fistula tract, which was permeated by the probe. Using two small retractors, diathermy and blunt dissection could be successfully utilized to meticulously dissect the intersphincteric plane to the level of the probed fistula tract. After gentle separation of the internal and external sphincters, it was possible to encircle and isolate the tract. Two absorbable (3/0 polyglycolic acid) sutures were used to secure the fistula tract as close as possible to the lateral margin of the internal anal sphincter and the medial margin of the external anal sphincter. After double suture-ligation of the fistula tract, it was divided between these two sutures. No specimen was sent to pathologic examination. The intersphincteric plane was then irrigated with saline, revised for hemostasis, and closed in two layers (muscle approximation and skin) using interrupted 3/0 polyglycolic acid suture. The skin incision was glued with Dermabond^®^ (Ethicon Inc., Cincinnati, OH, USA) at surgeons’ discretion. Orifice excision at the external opening was undertaken to facilitate drainage and prevent early closure of the distal tract.

### Postoperative care

All procedures were performed in an inpatient setting. Patients were discharged with prescription of anti-inflammatory agents, narcotic analgesics, and stool softeners in the next morning. They were instructed to use sitz baths two times daily and always after a bowel movement. Patients were routinely reviewed in the outpatient clinic two weeks after surgery. Subsequent office follow-up was scheduled at 2- to 4-week intervals until clinical diagnosis of healing. For patients with suspected recurrence, radiologic confirmation using MRI was mandatory. Due to the preparation of the present manuscript, all included patients were clinically re-evaluated in order to rule out recurrence and to define precise follow-up duration. 

## RESULTS

Between November 2015 and November 2017, 38 consecutive patients with newly diagnosed or recurrent anal fistulas were operated on using the LIFT technique. Seventeen (44.7%) were male. Median age was 41 (range, 18-67) years. Median BMI was 26.4 (22-38) kg/m^2^. In seven (18.4%), one or more comorbidities were observed. Four (10.5%) patients had hypertension and three (7.9%) were diabetic. The median distance between the external orifice and the anus was 5 cm (range, 3-8). Seventeen (44.7%) patients had undergone preoperative MRI scan. Five (13.2%) to a previous sphincter-sparing operation (excluding abscess incision and drainage) for anal fistula. Therefore, in this series, five (13.2%) patients have undergone the modified LIFT procedure due to recurrence. In three (8%) cases, the prior procedure was the anal fistula plug (Biodesign^®^ Fistula Plug Set, Cook Medical, Bloomington, IN, USA) and in two (5.2%), the video-assisted anal fistula (VAAFT^®^, Karl Storz, Tuttlingen, Germany) treatment^19^. In the present series, the fistula was transsphincteric in all cases (Table 1). Operative and early postoperative results are showed in Table 2. There were no intra- or postoperative complications. 

**TABLE 1:** Patient demographics

Demographic data	N (% or range)
Number of patients	38
Male gender (%)	17 (44.7)
Median age (years)	41 (18-67)
Median BMI (kg/m2)	26.4 (22-38)
Comorbidities (%)	7 (18.4)
Median distance between external orifice and the anus (cm)	5 (3-8)
Previous sphincter preserving surgery for anal fistula (%)	5 (13.2)
Type of fistula (%) Transsphincteric	38 (100)

**TABLE 2:** Operative and postoperative results

Variable	Value (% or range)
Operative time (min)	30 (20-45)
Median follow-up (weeks)	32 (14-56)
Success after LIFT (%)	30 (79)
Median time interval to recurrence (weeks)	24.8 (18-42.5)
Median follow-up (weeks) after surgical treatment of failures	6 (3-7)

### Success

No patients were lost to follow-up. Median follow-up duration was 32 weeks (range, 14-56). 

Success was observed in 30 (79%) patients. At the end of the present follow-up, eight (21%) presented with recurrence after LIFT. Three of these had already undergone a sphincter-preserving fistula surgery (anal fistula plug in one case; and the VAAFT^®^ technique in the remaining case). For two patients in the present series previously submitted to a sphincter-preserving technique, success was observed after the LIFT procedure. 

Regarding the eight patients with recurrent fistula after LIFT (failures), the median time interval to recurrence was 24.8 weeks (range, 18-42.5). In all failure cases, the recurrent external orifice was observed at the LIFT surgical incision scar. Therefore, all recurrent fistulas became intersphincteric. In the present study, all eight failures were successfully reoperated on using a single fistulotomy (lay-open technique) approach. The mean follow-up after the successful lay-open technique for failures in the present study was six weeks (3-7).

## DISCUSSION

In this bi-institutional Brazilian experience with the LIFT procedure, it was observed that 79% of patients with anal fistulas not amenable to surgery through a simple fistulotomy were cured after an isolated LIFT procedure without previous seton drainage. Moreover, the operation proved itself safe and easy to learn. Ultimately, since no specialized or expensive materials like laser, collagen plugs or video-assisted devices were used; the procedure represents an attractive option for operating on anal fistula patients in under-development countries. 

At least six different technical variations of the originally described LIFT procedure have been identified in the literature^8^. Since no direct comparison between the technical variations has been done yet, it remains challenging to ascertain the true efficacy of the classic LIFT or any of its technical variations^16^. The LIFT technique without excision of the fistula tract, which represents a slight variation of the original procedure^7^ was used in all patients operated on in the present study. As other have stated, it is our belief that the success after anal fistula surgery derives from proper identification and handling of the internal opening, instead of what can be done about the tract itself. A success rate from 47-94.2% has been reported with the use of this variation of the LIFT technique^12^ ^,^ ^20^ ^-^ ^23^.

Currently, there might be some controversy regarding the definitions of failure, persistence, and recurrence after anal fistula surgery. Anyway, success after the LIFT procedure may be objectively defined as complete postoperative healing of both the original external fistula opening and the surgical access (incision). Conversely, failure may be defined as persistent discharge through the original fistula external opening or the intersphincteric wound. Finally, recurrence may be considered as the reappearance of fistula drainage after complete wound healing^6^. Conceptually, it is probably true that all failures involve the intersphincteric wound, and all the recurrences must have a tract from the previous internal opening to an external opening. However, we believe that, oftentimes, it might be a tough assignment to clinically discern between persistence and recurrence. One should consider a case where the surgical wound evolved without drainage for a week. After this time interval, a purulent drainage starts draining through the incision. We would be facing persistence or recurrence? In the present study, we have decided to simply rank the case as a failure.

Some authors have suggested the use of a seton drainage prior to the LIFT procedure as an effective tool against failure after surgery, although it was not mentioned in the original technique descriptive publication^7^. In the present study, the use of a pre-LIFT seton drainage was never used. Three case-series reporting on predictive factors associated with success after LIFT for transsphincteric fistula have shown that the use of pre-LIFT drainage seton did not affect success rates^11^ ^,^ ^14^ ^,^ ^15^. Moreover, in an analysis of pooled data from four studies of patients undergoing LIFT with and without preoperative drainage seton, no significant difference (RR 0.96; 95 % CI 0.8-1.16; p=0.69) associated with drainage could be demonstrated^16^. Notwithstanding, these results should be interpreted with caution, since in the three case series, the initial study design was not directed to investigate or compare the outcomes of patients with and without seton placement. 

Few authors have succeeded when reporting on other risk factors for failure after the LIFT procedure or its variations, thus preventing the initiative of tailoring the surgical procedure to a given patient’s risk profile. Abcarian et al.^13^ have published the negative impact of the number of previous fistula operations over the success rates after LIFT. Conversely, Wallin et al.^11^ and Liu et al.^14^ were not able to prove an association between the number of previous fistula operations and success of LIFT. In addition, Liu et al.^14^ could not demonstrate an association between a fistula tract of less than 3 cm and a higher healing rate following the LIFT procedure. Unfortunately, the present series was to small to enable a proper analysis of risk factors associated to failure. However, still on the subject of failure after LIFT for anal fistula, two important aspects should be featured. First, most studies had limited follow-up. Therefore, more experiences are needed with longer follow-up periods. Second, when failure was diagnosed after LIFT, it was possible to convert an original transsphincteric fistula to either an intersphincteric sinus or fistula, therefore simplifying surgical management. In the present study, all failures could be successfully managed through a simple fistulotomy (lay open technique) operation.

The main limitations of this study come from its non-comparative design, from a limited follow-up, and from the absence of evaluation regarding variables associated with failure after LIFT. First, the surgeons of the present study group had already experienced other sphincter-sparing techniques such as the mucosal advancement flap (most commonly), and the anal fistula plug among with the VAAFT technique^24^ more occasionally. Based on the concept that preliminar evidence suggests that the mucosal advancement flap has a longer operative time and delayed associated recovery^21^ and the collagen plug results in an unsatisfactory cure rate^25^ ^,^ ^26^, we decided to undertake this bi-institutional prospective early experience with the LIFT technique in a non-comparative manner. Our experience, among others, continue to provide more information regarding the LIFT procedure. However, further evidence is still necessary to determine the long-term outcome of this technique, its impact on continence, and how it compares with other sphincter-sparing operations. Finally, due to a limited number of cases, cause-and-effect relationships between clinical variables and failure could remain undetected, or even be false. 

Further studies are demanded to identify risk factors for treatment failures and effectiveness of LIFT compared to other sphincter-preserving operation for anal fistulas.

## CONCLUSION

The LIFT technique without excision of the fistula tract proved to be safe and effective for transsphincteric anal fistulas.
